# Nanomedicines for the Delivery of Biologics

**DOI:** 10.3390/pharmaceutics11050210

**Published:** 2019-05-03

**Authors:** John Wahlich, Arpan Desai, Francesca Greco, Kathryn Hill, Arwyn T. Jones, Randall J. Mrsny, Gianfranco Pasut, Yvonne Perrie, F. Philipp Seib, Leonard W. Seymour, Ijeoma F. Uchegbu

**Affiliations:** 1 The Academy of Pharmaceutical Sciences, 4 Heydon Road, Great Chishill, Royston SG8 8SR, UK; 2 Advanced Drug Delivery, Pharmaceutical Sciences, IMED Biotech Unit, AstraZeneca, Granta Park, Cambridge CB21 6GH, UK; Arpan.Desai@astrazeneca.com; 3 Reading School of Pharmacy, University of Reading, Whiteknights, Reading RG6 6AP, UK; f.greco@reading.ac.uk; 4 Global Product Development, Pharmaceutical Technology and Development, Operations, AstraZeneca, Macclesfield SK10 2NA, UK; Kathryn.Hill@astrazeneca.com; 5 Cardiff School of Pharmacy and Pharmaceutical Sciences, Cardiff University, Cardiff CF10 3NB, UK; JonesAT@cardiff.ac.uk; 6 Department of Pharmacy and Pharmacology, University of Bath, Bath BA2 7AY, UK; r.j.mrsny@bath.ac.uk; 7 Pharmaceutical and Pharmacological Sciences Department, University of Padova, F. Marzolo 5, 35131 Padova, Italy; gianfranco.pasut@unipd.it; 8 Strathclyde Institute of Pharmacy and Biomedical Sciences, University of Strathclyde, Glasgow G4 0RE, UK; yvonne.perrie@strath.ac.uk (Y.P.); philipp.seib@strath.ac.uk (F.P.S.); 9 Department of Oncology, Old Road Campus Research Building, Oxford OX3 7DQ, UK; len.seymour@oncology.ox.ac.uk; 10 UCL School of Pharmacy, London WC1N 1AX, UK; ijeoma.f.uchegbu@pharmacy.ac.uk

**Keywords:** nanomedicines, drug delivery, siRNA, mRNA, DNA, proteins, lipid nanoparticles, liposomes, viral vectors, endocytosis

## Abstract

A special symposium of the Academy of Pharmaceutical Sciences Nanomedicines Focus Group reviewed the current status of the use of nanomedicines for the delivery of biologics drugs. This meeting was particularly timely with the recent approval of the first siRNA-containing product Onpattro™ (patisiran), which is formulated as a lipid nanoparticle for intravenous infusion, and the increasing interest in the use of nanomedicines for the oral delivery of biologics. The challenges in delivering such molecules were discussed with specific emphasis on the delivery both across and into cells. The latest developments in Molecular Envelope Technology^®^ (Nanomerics Ltd, London, UK), liposomal drug delivery (both from an academic and industrial perspective), opportunities offered by the endocytic pathway, delivery using genetically engineered viral vectors (PsiOxus Technologies Ltd, Abingdon, UK), Transint™ technology (Applied Molecular Transport Inc., South San Francisco, CA, USA), which has the potential to deliver a wide range of macromolecules, and AstraZeneca’s initiatives in mRNA delivery were covered with a focus on their uses in difficult to treat diseases, including cancers. Preclinical data were presented for each of the technologies and where sufficiently advanced, plans for clinical studies as well as early clinical data. The meeting covered the work in progress in this exciting area and highlighted some key technologies to look out for in the future.

## 1. Discussion

The Academy of Pharmaceutical Sciences (APS, the professional body for Pharmaceutical Scientists in the UK) hosted a meeting of its Nanomedicines Focus Group at the University of Reading on 18th July 2018 on the challenges of delivering biological molecules and in particular the role of nanomedicines. The meeting heard from some of the EU’s leading experts on the recent developments in this exciting area of the pharmaceutical sciences.

The global market for biologics drugs is extremely large with eight out of the current 10 best-selling pharmaceutical products being proteins (six are monoclonal antibodies and one a vaccine) [1]. Forty-five biologics drugs have reached blockbuster status (sales over $1Billion) [2]. Sixty-two proteins were approved by the US Food and Drug Administration (FDA) between 2011 and 2016 [3] and FDA Centre for Drug Evaluation and Research (CDER) currently lists 150 approved biologics (including biosimilars) [3]. 

In addition to peptides and proteins, there is an increasing interest in the delivery of nucleic acid-based therapeutics (DNA, siRNA, mRNA etc.) that in turn have the ability to fine-tune the cellular response. First generation siRNA therapeutics are now entering routine clinical use (patisiran, an antisense oligonucleotide for the treatment of the polyneuropathy, Alnylam Pharmaceuticals, Inc) demonstrating that delivery challenges can be overcome. Of further interest is the delivery of viruses which could have the added benefit of replicating at the target site hence amplifying their biological payload. 

The administration of biologics drugs, however, remains a significant challenge. The 7 leading biologics drugs mentioned (in recent sales order: Humira^®^, Eylea^®^, Rituxan^®^, Enbrel^®^, Herceptin^®^, Avastin^®^ and Remicade^®^) are all delivered parenterally. In some cases, this requires a hospital visit or multiple injections and this can be inconvenient for both the practitioner and the patient. Hence, the increasing interest in the oral delivery of biologics [4].

Biologics drugs are challenging to deliver because, apart from failing to meet Lipinski’s ‘rule of five’ criteria, the large molecules tend to be unstable both in vitro requiring care in formulation to prevent aggregation etc. and in vivo due to chemical and enzymatic degradation. For many biologics drugs, one of the biggest challenges is delivering these molecules across the cell membrane to their intracellular target and the avoidance of intracellular degradation pathways. Oral delivery of biological drugs, which could significantly improve patient convenience, presents huge challenges due to the additional hurdles of the unfavourable environment within the gastro-intestinal (GI) tract and difficulty in delivering macromolecules across the gut epithelium and into the systemic circulation. 

Molecules can cross the epithelium either by passage through the enterocyte (transcellular route) or through the tight junctions between the enterocytes (paracellular route). Neither route is well suited to large molecular weight molecules. The paracellular route is very restricted due to the low frequency of the tight junctions and the limited size of molecules that can pass through (the tight junction is generally impermeable to molecules of radius greater than approximately 11–15 Å, which is too small for most polypeptides and certainly proteins [5]. The transcellular route mainly via endocytosis, therefore, offers the better prospect of transport across the membrane (for an excellent review see Reference [6].

**Figure 1:**
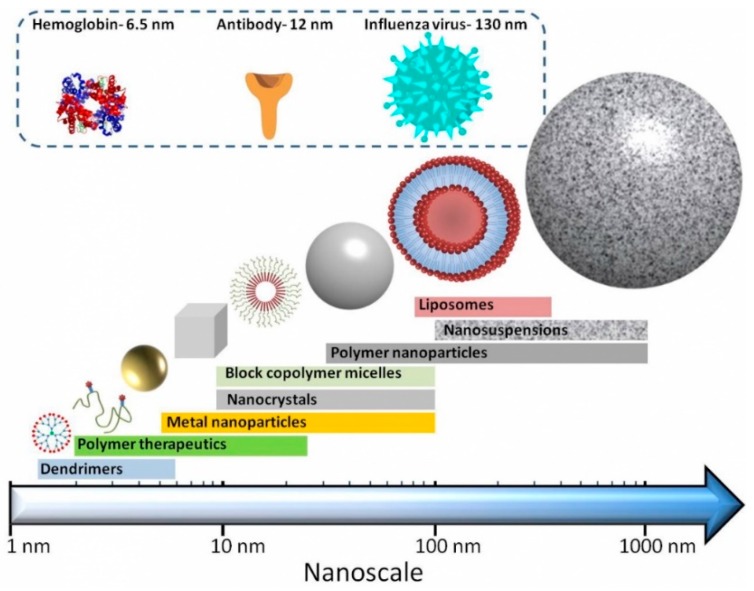
Cartoons of nanomedicines and their approximate sizes compared to various biological molecules (reproduced with permission from the British Society for Nanomedicine: https://www.britishsocietynanomedicine.org/what-is-nanomedicine/ (accessed on 10 October 2018)). Note that lipid nanoparticles can vary in size from 50–1000 nm.

Nanosized drug delivery systems ‘nanomedicines’ (see Figure 1) are one strategy to improve the delivery of biologics drugs. They fall into broad categories, including (i) nanoparticles (e.g., liposomes, where the delivery system physical encapsulates the active molecule), (ii) drug-conjugates (e.g., antibody-drug conjugates, polymer-drug conjugates, where the active molecule is chemically conjugated to the delivery systems), (iii) protein-polymer conjugates and (iv) combinations thereof. 

Nanomedicines have several advantages [7]: their size and surface characteristics can be manipulated; they can control and sustain the release of the payload at the target site; drug loading may be sufficiently high to balance the benefits of the active pharmaceutical ingredient (API) with the costs and potential side effects of the excipients; controlled release and carrier degradation characteristics can be modulated; site-specific targeting can be achieved by attaching targeting ligands to the surface; and they can be used for various routes of administration including oral, nasal, intra-ocular etc. in addition to the parenteral route. The use of nanomedicines in cancer therapies has received particular attention [8] due mainly to their potential to improve drug therapeutic index.

The presentations at this conference spanned a broad range of topics from insights into the mechanisms of cellular uptake and new synthetic and screening approaches to aid nanomedicine design to preclinical data for a broad range of emerging nanomedicine technologies for biopharmaceutical delivery and scale-up considerations. The following is a summary of the presentations.

Ijeoma Uchegbu (University College London, School of Pharmacy, UK; Chief Scientific Officer of Nanomerics, London, UK) reported on recent work [9] where her group developed nanoparticles (50–450 nm) made from *N*-(2-ethylamino)-6-O-glycolchitosan (EAGC) for the delivery of siRNA or DNA which, by limiting the level of tertiary amines on the polyamine, was 10 to 50-fold less toxic than Lipofectamine 2000 a common liposomal transfection reagent. The higher biocompatibility allowed 10-fold higher doses of DNA and siRNA to be applied to cells in vitro and intra-nasal dosing to rats resulted in siRNA transfer to the olfactory bulb. However, they subsequently concluded that the nanoparticles were not efficient enough as a gene delivery system. 

Professor Uchegbu went on to describe the Molecular Envelope nanoparticle Technology being developed in Nanomerics Ltd a spin-out company from University College London founded in 2010. Work by Ahmad et al. [10] showed how, using drug-polymer modelling, a chitosan amphiphile consisting of quaternary ammonium palmitoyl glycol chitosan was able to bind propofol (as a model drug). Subsequent work showed how palmitoyl glycol chitosan nanoparticles were able to increase the oral bioavailability of cyclosporine by 100% compared to the marketed Neoral^®^ product [11]. The increase was due to a triple mode of action with the nanoparticles (i) increasing the dissolution of the hydrophobic active pharmaceutical ingredient, (ii) adhering to and penetrating the mucus layer thus enabling intimate contact between the drug and the epithelium, and (iii) enhancing transcellular transport. 

The molecular envelope system with the palmitoyl glycol chitosan polymer produces very stable self-assemblies of nanoparticles which have a high ∆G, a consequence of their cone-shaped architecture. They have a high surface area with hydrophobic groups on the surface with their self-assembly being driven by the entropy change produced by the release of water molecules. Their critical micellar concentration was found to be 19 μM and they can accept up to a 50% *w*/*w* drug loading. Oral particle uptake of amphotericin B by epithelial cells and translocation to specific organs has been observed in models for visceral leishmaniasis, candidiasis and aspergillosis [12]. Toxicity testing of the palmitoyl glycol chitosan polymer has been completed and several drugs are in early-stage development using this technology.

The compound NM0127 under development by Nanomerics Ltd uses the Molecular Envelope Technology^®^ together with an endogenous Leucine5- enkephalin (LENK) peptide for the potential treatment of breakthrough cancer pain as an alternative to opiates. This potential product aims to target the central nervous system. As 95% of drugs fail to target the central nervous system as they are excluded by the blood-brain barrier this is a major challenge. Initial work by Mazza et al. [13] used dalargin (another leucine containing enkephalin) and showed that if it was injected ‘as is’ the drug was below the level of detection in any of the assessed organs. When linked to a palmitoyl moiety via an ester linkage it self-assembled into nanofibres (acting as a pro-drug) and when injected into mice, dalargin was detected in all examined organs. It also gave a positive result in the mouse tail model of pain showing it affected the central nervous system by increasing the pain threshold. It appears that the nanofibre form protects the amphiphilic peptide from degradation while in the plasma, and the amphiphilic nature of the peptide promotes its transport across the blood-brain barrier. In follow on work a leucine^5^-enkephalin (LENK)-palmitoyl ester prodrug was generated and the resulting nanofibres (2 μm long by 2 nm wide) were coated with palmitoyl glycol chitosan [14]. The coating enabled the peptide prodrug to escape liver uptake and hence extended its plasma half-life by 520% and its brain bioavailability (i.e., area under the curve) by 47% as confirmed by the functional in vivo response in mice. 

However, for a commercial product for breakthrough pain, it is not practical to give the product by intravenous injection hence oral delivery of NM0127 was considered. However, oral bioavailability was found to be <1% for LENK. Therefore, the intra-nasal route was studied. The coated nanofibers [14] were formed into larger microparticles (20 μm) by spray drying to yield a product more suited to intranasal delivery [15]. The microparticles break down in the nasal cavity to yield the nanoparticles (20 nm) to achieve passage across the blood brain barrier. Intranasal dosing to rats showed activity in a pain model comparable to intravenous morphine and evidence of a central rather than a peripheral action. Remarkably, there was no evidence of tolerance after five days’ chronic dosing and surprisingly activity was found in morphine-tolerant animals albeit at a lower level [15]. Based on these pre-clinical studies Phase 1 clinical studies of NM0127 are planned that will use the intranasal dosing device from Alchemy Pharmatech Ltd. 

Yvonne Perrie (University of Strathclyde, Glasgow, UK) discussed the delivery of vaccines and highlighted that developing such products was a balancing act between their tolerability and their immunogenicity. Adjuvants are added to enhance the immune response to the vaccine [16]. Barocchi et al. [17] reviewed emerging technologies for the development of vaccines from the 1930s to the present day; most of the vaccines that have been in clinical trials since 2014 have used adjuvants [18]. In addition to the use of adjuvants, various nanoparticle delivery approaches have also been used to further enhance the immunogenic response [19].

There is an urgent need for a new tuberculosis vaccine that is safe, effective and affordable; protects against all strains of tuberculosis; is suitable for all ages and can be safely used in children living with HIV (where the current Bacillus Calmette-Guérin (BCG) vaccine is not recommended [20,21]). To tackle this unmet healthcare challenge Professor Perrie’s group have used liposomes to deliver the Ag85B-ESAT6 fusion antigen (a fusion protein from *Mycobacterium tuberculosis*) developed by the Statens Serum Institut (Copenhagen, Denmark). The liposomes consist of the cationic lipid dimethyldioctadecylammonium (DDA) together with the Toll-like receptor ligand/immunostimulant trehalose 6,6’-dibehenate (TDB) [22]. The TDB is incorporated into the DDA liposomal bilayers and effectively stabilises the liposomes. The cationic nature of the liposome facilitates the uptake of the negatively charged antigen.

Liposomes with and without TDB were manufactured with equivalent antigen loading (88%) [22]. They were approximately 416 and 488nm in diameter and had zeta potentials of approximately +48 and +46mV, respectively. Studies in infected guinea pigs showed efficacies for both types, comparable to BCG with the liposomes acting as both delivery agent and adjuvant in the absence of TDB. Biodistribution studies were performed following two or three immunisations 28 days apart by intramuscular injection in mice. To trace both the carrier and the antigen radiolabelling was used (i.e. antigen with 125I and the liposome with 3H). Liposomes promote a depot effect while the TDB promoted monocyte recruitment. 

There has been concern over the toxicity of cationic liposomes [23] therefore liposomes with a neutral lipid (DSPC) were considered too. These were cleared faster from the mouse injection site with less monocyte influx indicating that the depot effect, seen with the cationic liposomes, was important for activity. Of more importance than the uptake of the antigen is the production of Interferon-gamma (IFNγ, a cytokine indicative of the generation of an immune response as it is an inducer of the IgG2a antibody) [24]. An increase in IFNγ was noted as the amount of DDA in the liposome increased and the amount of DSPC decreased while keeping TDB at a constant level. An even greater response (as monitored by an increase in IgG2a antibodies) was detected if the injection was made directly into the lymph node [25] but this is not a clinically practical route of administration. 

Is it possible to retain the depot effect but hide the charge of the liposomes? PEGylation of the liposome lowered the zeta potential while retaining the antigen load. At 10% PEGylation (120 nm liposomes) the depot effect was maintained, and monocytes were recruited. At 25% PEGylation liposomes travelled from the injection site to the lymph nodes which boosted early antibody response but produced lower IFNγ levels demonstrating the importance of the depot effect. 

Resiquimod is a small molecule immune response modifier which acts as an agonist on the endosome-located Toll-like receptor 7. Resiquimod was conjugated to both the DSPE and the DDA:TDB liposome [26]. Resiquimod remained with the liposome but upon exciting the injection site increased the production of IFNγ in the spleen (but resiquimod simply mixed with the DDA:TDB liposome has a negligible effect). 

The liposomal vaccine formulation is of little benefit if it cannot be manufactured at scale. On a laboratory scale, liposomes are routinely made using a rotary evaporator. This process is very difficult to scale up. The problems of liposome manufacture came to a head in 2011 when a shortage of Caelyx^®^ (liposomal doxorubicin) was reported due to manufacturing problems.

Professor Perrie reported how her group has been working with the Precision NanoSystems’s Inc. NanoAssemblr^®^ equipment, which uses a flow-through microfluidic cell to manufacture liposomes, a process which is easily scaled up by modifying the flow and increasing the number of cells. Streams of lipids/polymers (in solvents) and of drug/antigen (aqueous) are combined in the herringbone-channelled cell [27] to produce liposomes within minutes. Critical process parameters (including lipid formulation, lipid concentration, flow rate ratio) and the critical quality attributes of the liposomes (including size, polydispersity, lipid yield, loading and release characteristics) were studied. For example, the effect of the flow rate ratio (lipid flow to aqueous flow) impacted liposome diameter while their polydispersity index was dependent on the formulation of neutral and cationic liposomes [28]. 

The solvent used to dissolve the lipid (acetonitrile has been used) can be removed by tangential flow filtration. Lipid recovery (important as the lipid is expensive) is of the order of 95%. Further work is planned with Malvern Instruments Ltd to develop ‘at line’ particle sizing of the liposomes and the next step will be to automate the quantification of drug/antigen loading. 

Gianfranco Pasut (University of Padua, Italy) described the difficulty of delivering proteins with the challenges including chemical and physical instability, enzymatic degradation, aggregation, rapid kidney clearance and immunogenicity. As a consequence, proteins may require frequent administration and high doses greatly exacerbating their side effects and reducing patient compliance. Conjugating the protein with a polymer reduces their clearance and can alter their biodistribution (any loss of activity being compensated for by an increase in half-life). In addition, conjugation may affect aggregation, reduce immunogenicity and improve the stability of the protein to proteolytic enzymes. Today there are 16 PEGylated proteins on the market plus one biosimilar of pegfilgrastim (Fulphila™).

Cimzia^®^ (certolizumab pegol), Jivi™ (PEGylated Factor VIII), Rebinyn® (glycoPEGylated coagulation factor IX), Lonquex® (lipegfilgrastim), Neulasta® (pegfilgrastim) and Plegridy® (PEGinterferon beta 1a) are all linked 1:1 and site selectively with a PEG molecule while others (e.g., Somavert® pegvisomant) are randomly PEGylated at different sites in the protein, thus generating a conjugate composed by a mixture of isomers. Both the site of PEGylation and the stability of the linker are important. Attachment to a lysine amino acid is most common but linkages can be to cysteine thiol group, to cysteine-cysteine disulphide bonds after reduction or to N-terminal amino groups. 

Site-selective attachment of polymers to proteins is desired but thiol conjugation to free cysteine is difficult owing to their typically low accessibility and, therefore, N-terminal linkage remains the only affordable possibility but is well covered by patents. A new option is the use of an enzymatic method of conjugation to form these protein-polymer covalent bonds, exploiting the advantage of enzyme selectivity and ability to perform the reactions at physiological pHs with good conversion yields. GlycoPEGylation is one example where PEG chains are conjugated to protein glycans without destroying the sugar structure. Rebinyn^®^ is an example in which the PEGylation of the Factor IX protein lengthens the half-life of the protein in the blood. 

Transglutaminase (TGase) is another enzyme well suited to selectively mediate polymer conjugation at specific glutamines but, as shown recently, also to lysine [29]. There are several transglutaminases from different sources with different site specificities and they work via a single step reaction on many native proteins without the need to add specific substrate sequences [30]. However, the glutamine must be in a “flexible” peptide sequence to adapt to the enzyme catalytic site to permit polymer conjugation. Importantly, the TGase conjugation strategy results in a heterogeneous product when there is more than one (flexible and accessible) glutamine. However, the flexibility can be manipulated to achieve specificity by changing the solvent used for the reaction [31]. By considering the positioning of the glutamine it is possible to direct binding to a region not implicated in the therapeutic interaction (and thus preserving the biological activity of the conjugate). The complicating factor is that transglutaminase is in solution and thus needs to be separated from the product. The transglutaminase can be immobilised which aids product separation and minimises subsequent reaction with the PEGylated product.

An alternative approach is to add the glutamine to the PEG to direct polymer conjugation to a lysine on the protein (also requiring flexibility). In addition, in this case, the requirement of flexibility is important. This reaction can be exploited to add other polymers to a protein as well [29]. 

There has been some concern about the toxicity of PEG due to the presence of anti-PEG antibodies in humans [32]. However, the significance of this, in terms of safety is highly debated in the scientific community and is likely less of a concern than the immunogenicity of the protein itself. Should this prove to be wrong alternative polymers candidates are available; for example, hyaluronic acid (a biodegradable biopolymer). Hyaluronic acid receptors are abundant in some specific tissues such as liver, kidney, and most tumour tissues opening further targeting opportunities [33]. 

Arwyn Jones (Cardiff University, UK) described the endocytic gateways for macromolecules to enter cells. The routes regulated by clathrin are best characterised but other, non-clathrin mediated endocytic (uptake) routes exist [34]. For cells to maintain and regulate cellular volume, plasma membrane composition and respond to environmental cues there is a fine-tuned balance between endocytosis (i.e., uptake) and exocytosis (efflux). One regulatory mechanism of exocytosis is exosomes that can be sent off into the extracellular environment. Exosomes are small (30–100 nm) membranous nanovesicles (akin to nano-sized liposomes) which are products of the endocytic machinery. These exosomes comprise a lipid bilayer membrane plus membrane-associated proteins and have been shown to regulate several functions, for example facilitating cell to cell communication [35]. There is considerable interest in exploiting exosomes as a “biologic drug delivery systems” because one of their inherent functions is to deliver macromolecular cargoes. Roberts-Dalton et al. [36] describes a new method of labelling thiol groups on exosomes to allow characterisation of their endocytic uptake and subcellular fate and future studies on their ability to influence recipient cells. 

Endocytosis can traffic (macromolecular) payloads into many cellular locations including endocytic structures (e.g., late endosomes) that can mature into lysosomes where a combination of low pH and (proteolytic) enzymes can degrade the payload. The harsh lysosomal environment is detrimental for many biologics and has therefore been avoided. Instead, sophisticated nanomedicines have been designed with the aim to achieve cytosolic escape of the payload from endosomes. Nonetheless, lysosomal-mediated drug delivery (i.e., lysosomotropic, [37]) has traditionally been extensively explored for small molecular weight anticancer drug delivery [38]. New paradigms for targeting the lysosome are emerging (especially to induce receptor down-regulation). For example, delivery to this organelle enables either degradation of oncogenic receptors that are overexpressed in cancers or release of prodrugs from antibody–drug conjugates. Moody et al. [39] exogenously added biotinylated proteins or antibodies to cells and optionally induced cross-linking with streptavidin. Importantly this cross-linking rewired the ligand specific endocytic pathway to the lysosomal route. Moody showed (via confocal microscopy) that the delivery of three different proteins (transferrin, an iron-transporting protein) and two antibodies (anti-MHC Class 1 antibody W6/32) and the clinically approved anti-HER2 antibody trastuzumab (Herceptin^®^) could now be readily targeted to lysosomes. In the absence of crosslinking, transferrin (an extensively recycled ligand) was recycled back to the plasma membrane and released into the extracellular environment instead of trafficking into lysosomes. The anti-MHC antibody locates to the plasma membrane and is only slowly internalised to endosomes and lysosomes over 4 h. However, in the presence of crosslinking the anti-MHC antibody was rapidly depleted from the plasma membrane (30 min) to vesicles showing extensive colocalization with lysosomes after 4 h. The HER2 antibody is regarded as the ‘endocytosis-resistant’ member of the ErbB family of receptors proving problematic for its targeted down-regulation and use as a target for antibody–drug conjugates. In the presence of crosslinking there is dramatic internalisation of Herceptin^®^ to endocytic structures and lysosomes where it is then degraded [39,40].

The exact endocytic pathway followed by HER2/Herceptin^®^ complexes (e.g., whether it is or is not clathrin-mediated endocytosis or clathrin-independent) has not been elucidated hence the abbreviation NIE for ‘No Idea Endocytosis’ has been coined! Scanning electron microscopy showed dramatic responses to clustering and the link between this and enhanced endocytosis is still under scrutiny. Notably, resistance towards Herceptin^®^ is a clinical challenge [41] and whether cross-linking of the antibody could influence the resistance is currently under investigation.

Professor Jones discussed recent research on the targeting of plasma membrane receptors using responsive polymer nanoparticles. Work by Brazzale et al. [42] examined active targeting of cells for intracellular delivery. They used gold nanoparticles decorated with two polymers (a 2KDa g/mol PEG with a terminal folate targeting ligand and a di-block copolymer including a pH-responsive and a hydrophilic block). At the serum pH of 7.4, the pH-responsive block (apparent pKa 7.1) showed an extended conformation shielding the PEG-folate ligands and inhibiting cellular uptake. Under pH conditions resembling those of the extracellular matrix around solid tumours (approximate pH 6.5), the di-block copolymer collapsed exposing the folate residues terminally conjugated to the PEG chains. The presence of the folate residues facilitated receptor-mediated endocytosis exploiting folate receptor overexpression in certain cancer cells.

The second piece of work [43] characterised how receptor targeting agents on nanoparticles interact at cell surface receptors and whether it is possible to control these interactions via exogenous stimuli. In this case transferrin at the surface of thermoresponsive polymer-coated gold nanoparticles was studied. Varying the temperature had the effect of sterically ‘hiding’ the transferrin if the polymer chain was extended or ‘revealing’ it if the polymer chain was collapsed. Once again there was strong evidence from live cell imaging confocal studies showing that transferrin and transferrin receptor endosomal traffic was being diverted to lysosomes rather than entering recycling pathways.

Tam et al. [44] reviewed the use of lipid nanoparticles for siRNA delivery; some cells were easy to transfect, and others were harder (e.g., primary blood and leukaemia cells, [45]). mRNA delivery to different cell types using lipid nanoparticles was discussed with the aim of identifying key endocytic features that influence transfection. Work on comparative high content endocytic analysis of different cell lines incubated with fluorescent (mRNA) lipid nanoparticles highlighted some key features that may affect delivery to the cytosol and translation to protein [43].

Arpan Desai (AstraZeneca, UK) described the research activities underway in AstraZeneca’s newly formed Advanced Drug Delivery Group. AstraZeneca’s work on antisense oligonucleotides (which decrease protein expression) is in collaboration with Ionis Pharmaceuticals Inc.: clinical targets in oncology include the STAT3 transcription factor targeted by AZD9150 (in head and neck cancer NCT02499328). AstraZeneca is also collaborating with Moderna Therapeutics Inc. and Ethris GmbH on the delivery of mRNA for expression of therapeutic proteins. Examples of mRNA targets of interest are IL-12 and vascular endothelial growth factor (VEGF), which are being pursued as part of the Moderna Therapeutics Inc. collaboration. Several administration routes for biological drugs are being investigated. The most attractive option in the long term is oral delivery. However, a more realistic goal in the short term is a move from intravenous infusion to subcutaneous administration.

The antisense oligos are chemically modified to improve pharmacokinetic properties, eliminating special formulation requirements for intravenous administration. However, mRNAs, in contrast, require advanced formulations and a delivery system [46]. The immunogenicity of mRNA molecules has slowed clinical translation, but this has been overcome by modifying some of their bases [47]. The challenge is their delivery. The oligonucleotide mRNAs are >300 kDa, negatively charged and are susceptible to degradation. It is not possible to chemically modify them (as with antisense oligos) as this hinders protein translation. Naked delivery of mRNA is possible but is limited to local delivery to targets requiring a low degree of protein expression (VEGF) can be delivered by intra-cardiac injection for example). 

There are approximately 15 clinical trials in progress with mRNAs [48] and of these, 12 use naked delivery and three nanoparticles (for Zika virus NCT03014089; advanced melanoma NCT02410733 and triple negative breast cancer NCT02316457). AstraZeneca’s efforts on mRNA research are focussed in the following three therapeutic areas, where they are exploring therapeutic strategies involving local and systemic administration routes:

- Oncology
- Respiratory, inflammatory and autoimmune disorders
- Cardiovascular, renal and metabolic diseases

Lipid nanoparticles appear to have been most effective as delivery carriers for oligonucleotides [6]. Rozmanov et al. [49] have extensively characterised the lipid nanoparticles and earlier work by Akinc et al. [50] studied the mechanism by which they enter cells. Pieter Cullis’s group at the University of British Columbia in Vancouver, Canada [51] reports on the use of lipid nanoparticles for siRNA delivery with a product (developed with Alnylam Pharmaceuticals Inc.) submitted to the FDA in 2017 Lipid nanoparticle Onpattro^®^ (patisiran) containing siRNA for the treatment of polyneuropathy was approved by the FDA and EMA in August 2018. 

The inefficient endosomal release of lipid nanoparticles containing mRNA (the lipids are neutral at physiological pH and positively charged in the endosome which facilitates their fusion with and rupture of the endosomal membrane) means that in many cases, the amount of lipid required exceeds the toxic dose. AstraZeneca has explored how to widen the therapeutic window of lipid nanoparticles and has used high throughput screening to identify small molecules that will enhance lipid nanoparticles—mRNA delivery. They found that there was a huge variation in how different cell types are transfected with lipid nanoparticles and that there was typically no correlation between uptake and transfection (as compounds may enter the cell through an endocytic pathway but are then not released from the endosomes). Transfection rather than uptake is the critical measure of an effective enhancer molecule. The screening not only gave insights into the lipid nanoparticles uptake mechanism but also leads to the possibility of a compound that might be co-formulated with lipid nanoparticles. 

After screening 2638 compounds they found 38 molecules which acted as enhancers and 10 of these improved the delivery to multiple cell types. Yang et al. [52] (Eshelman School of Pharmacy, University of North Carolina, Chapel Hill, USA) using a similar approach, identified the small molecule compound UNC10217832A as an effective enhancer for the delivery of antisense and siRNA in vitro and in vivo in mice.

Randy Mrsny (University of Bath, UK; and co-founder of Applied Molecular Transport, South San Francisco, California, USA) continued the theme of macromolecule delivery via endocytic pathways. His specific interest is the oral delivery of biopharmaceuticals where there are three key issues:

- Protection of the biopharmaceutical from the gastric environment
- Stabilisation and isolation from the enzymatic burden of the (upper) part of the small intestine
- Rapid trans-epithelial uptake and transport by an efficient transport mechanism

The first two issues can be tackled, respectively, using an enteric dosage form and targeting to the lower part of the small intestine which has a more neutral pH and reduced proteolytic activities compared to the stomach and duodenum. The third issue, however, remains a significant challenge. Addressing this last challenge was the focus of his talk.

Professor Mrsny highlighted that there are recycling endocytic pathways at the apical and basal sides of polarized epithelial cells and connecting these would provide a means by which macromolecules could pass across the epithelial cells and hence achieve transcytosis across an intestinal epithelial cell. He noted that certain pathogens can establish stable infections at the luminal surface of epithelial cells and studying their pathophysiological mechanisms led to the discovery of the cholix endotoxin isolated from *Vibrio cholerae* [53]. During the infection by certain sub-species of this pathogen, cholix undergoes transcytosis through a vesicular trafficking mechanism where this exotoxin hijacks several elements of the host cells and uses them as receptors through transient interactions. Once across the epithelium, the toxin enters local immune cells of the *lamina propria* through a surface-expressed scavenger receptor and traffics in these cells through a pathway that leads to their intoxication [54]. If the cholix protein is coupled with a dye, its rapid transfer across a membrane can be visualised. Various ways in which biopharmaceuticals can be coupled to non-toxic elements of the toxin to allow for their transcytosis and delivery to the *lamina propria* were described. To date, proteins, peptides, siRNA, and even 100 nm diameter beads have been successfully coupled to a cholix-based carrier and their transcytosis demonstrated in vitro and in vivo with high bioavailability [55]. 

Professor Mrsny, working with Applied Molecular Transport Inc., has now optimised the cholix-based carrier to retain the parts required for epithelial transport and omit any cytotoxic or immunogenic actions to create the so-called Transint™ carrier [56]. The first molecule being developed by Applied Molecular Transport Inc. uses the Transint™ carrier coupled with IL-10, an anti-inflammatory cytokine known for its potential to treat irritable bowel diseases. IL-10 itself when administered systemically to Crohn’s disease and ulcerative colitis patients was too toxic to achieve concentrations in the *lamina propria* that are required for therapeutic benefit. Transint™ AMT-101 was shown to reach the *lamina propria* but not enter the systemic circulation, providing the desired mechanism of local actions without systemic toxicity for this cytokine. In preclinical studies, inflammatory markers in irritable bowel diseases in animals were returned to normal. Importantly, the transcytosis mechanism hijacked by the Transint™ carrier functions in normal, healthy intestine and also in inflamed intestine [57,58]. 

Other compounds in development using the Transint™ carrier for local delivery to the intestinal *lamina propria* include IL-22 and an anti-TNFα molecule. Investigations of the mechanism for Transint™ transcytosis have identified the specific endogenous receptors hijacked by cholix in polarized intestinal epithelial cells. Certain cholix sequence variants have been identified with the capacity to retain efficient endocytosis but without the ability to complete transcytosis. These variants can be used to selectively target apical or apical and basal vesicular structures that might be useful for the delivery of RNA-based biopharmaceuticals. These cholix variants are now being considered for exploitation in drug delivery systems where selected organelles are the therapeutic target or where access to the cytoplasm through release from specific vesicles could be achieved (e.g., in various states of pre-cancerous hypertrophy [59]).

Len Seymour (University of Oxford, UK and scientific advisor to PsiOxus Therapeutics Ltd, Abingdon, UK) spoke about oncolytic viruses described as ‘weaponised nanomachines for targeted expression of anticancer biologics’. The delivery of viruses to cancer cells where they replicate in situ, lyse cells and can also express therapeutic proteins is seen as easier than delivering the proteins themselves. Liu et al. [60] described the history of oncolytic virotherapy. The first genetically modified oncolytic viral therapy Imlygic™ (talimogene laherparepvec), designed to replicate within tumours and produce an immunostimulatory protein called granulocyte-macrophage-colony-stimulating factor (GM-CSF), was approved in the EU and US in 2015 for the treatment of melanoma [61].

‘The Hallmarks of Cancer’ concept [62] describes the features that define a cancer. When a virus invades a cell, it causes some of the same consequences. By engineering a virus to remove some of these properties it will target cancer cells to fill some of the gaps. Enadenotucirev virus (EnAd) (under development by PsiOxus Therapeutics Ltd) is an example. Delivered intravenously, EnAd replicates only in tumour tissue and not within normal, non-cancerous tissue and is therefore anticipated to be capable of selectively destroying tumour cells and at the same time attracting cells of the immune system. Intravenous and intra-tumoural delivery of the virus in a Phase 1 study using patients with various epithelial cancers showed high local CD8+ cell infiltration in 80% of tested tumour samples, suggesting a potential EnAd-driven immune response [63]. 

The EnAD virus can be loaded with immuno-therapeutics to produce the so-called ‘Armed EnAd’. Antibodies or antibody fragments or BITEs have been incorporated. Bispecific T cell Engager (BiTE) technology is designed to bind polyclonal cytotoxic T cells and targeted malignant cells. The BiTE antibody construct is engineered from two flexibly linked single-chain antibodies. One antibody is specific for a selected surface antigen on targeted malignant cells and the other antibody is specific for CD3-tied to the T-cell receptor complex on the surface of T cells [64]. The results from BiTEs have been somewhat limited by (i) poor delivery kinetics and penetration into tumours, and (ii) on-target off-tumour activity, leading to dose-limiting toxicities [65]. Linking the production of BiTEs to oncolytic viral replication provides an exciting means to restrict production to the tumour site, widen their therapeutic window, and synergize with direct oncolysis.

EnAd BiTEs are being studied to target cancer-associated fibroblasts; 80% of a tumour’s mass is due to stroma cells supporting tumour growth and evolution. Fibroblasts are present and are critical contributors to pro-tumour microenvironment via chronic “wound-like” signalling. The BITE technology recognises the antigen on the fibroblasts and recruits T cells to kill them. 

Professor Seymour [66] has reviewed the contribution that European scientists have made to the impact oncolytic viruses will have in cancer treatment and highlighted the future promise for the technology. 

## 2. Conclusions

There are many nanomedicine technologies in preclinical and clinical development for the enhanced delivery of biologics drugs, including siRNA, mRNA, DNA and proteins. The recent FDA approval of Onpattro™ (patisiran), the first siRNA drug, which is formulated as an injectable lipid complex demonstrates the potential of this field. Presentations at this Academy of Pharmaceutical Sciences Nanomedicines Focus Group symposium provided snapshots on the latest research on uptake mechanisms by gut epithelial cells and cells at the site of action, formulation screening approaches and scale up considerations, all of which are aiding the development of more effective nanomedicines for biopharmaceutical delivery. Further presentations demonstrated the breadth of nanomedicine technologies for biologics delivery, which if successful clinically, offer new opportunities to target difficult to treat diseases.
